# Posthumous assisted reproduction and post-mortem paternity

**DOI:** 10.3389/frph.2026.1783702

**Published:** 2026-03-04

**Authors:** Costanza Raimondi, Simona Giardina, Simone S. Masilla, Pietro Refolo, Clara Todini, Antonio G. Spagnolo

**Affiliations:** 1 Department of Healthcare Surveillance and Bioethics, Section of Bioethics and Medical Humanities, Università Cattolica del Sacro Cuore, Rome, Italy; 2 Research Center for Clinical Bioethics & Medical Humanities, Università Cattolica del Sacro Cuore, Rome, Italy

**Keywords:** artificial reproductive technology, posthumous embryo transfer, posthumous reproduction, post-mortem embryo transfer, post-mortem reproduction

## Abstract

**Background:**

The advancement of artificial reproductive technologies (ART) has outpaced many existing legal and ethical frameworks, challenging foundational notions of parenthood, consent, and the temporality of reproductive decisions. Among the most complex developments is posthumous assisted reproduction. While medically feasible, this practice raises profound legal and ethical questions, especially regarding the nature and validity of consent to parenthood to a child who will be born to a deceased father.

**Aim:**

This article provides a comparative analysis of legislation and regulatory frameworks governing posthumous reproduction via embryo transfer (a topic less investigated compared to gamete retrieval) across selected European countries to contextualize this practice.

**Materials and methods:**

The study adopts a comparative methodology, analyzing laws, regulatory guidelines from several European countries: Belgium, France, Greece, Italy, Portugal, Spain, the Netherlands, United Kingdom. Sources include legal databases, national ART authority publications, and academic articles.

**Results:**

The analysis reveals a fragmented European landscape. France maintains a categorical prohibition on posthumous reproduction, while all other countries investigated permit it under different degrees of procedural and temporal safeguards, emphasizing explicit, written, and pre-mortem consent.

**Conclusions:**

Overall, posthumous reproduction is framed as a continuation of a parental project, but consent models and temporal limits vary, ranging from specific post-mortem authorization to reliance on prior ART consent alone. Most countries impose waiting periods of six to twelve months and temporal limits of one to five years, while the Netherlands applies the general ART age limit of forty-nine years, and Italy stands out for the absence of any time restriction.

## 1 Introduction

Over the past few decades, assisted reproductive technologies (ART) have evolved from experimental medical procedures into established instruments of reproductive medicine, deeply impacting contemporary understandings of fertility, family, and parental identity (1). Since the late 1970s, technological advancements *in vitro* fertilization (IVF), gamete donation, and cryopreservation have made it possible to overcome infertility, opening to new scenarios of parenthood. These developments have broadened the range of reproductive choices available to individuals and couples, while also generating unprecedented ethical questions (2).

Among the most complex of these challenges is posthumous assisted reproduction, which enables conception after the death of one partner. By allowing the continuation of a reproductive project beyond death, ART disrupts the natural temporality of procreation, transforming it from a shared biological and relational act into a decision that extends beyond life itself. This possibility forces societies and legal systems to confront difficult questions about autonomy, consent, and responsibility when the act of procreation is decoupled from the ongoing existence of its initiators.

The aim of this article is to examine how selected European jurisdictions regulate the practice of posthumous reproduction. This phenomenon may take three distinct forms: the use of cryopreserved gametes for fertilization after the death of one partner; the retrieval of gametes from a deceased partner; and the transfer of embryos that were fertilized while both partners were alive, following the death of one of them. This analysis focuses exclusively on the use of cryopreserved embryos following the death of the male partner, as the death of the female partner would necessitate recourse to surrogacy, an area that lies beyond the scope of the present study.

The research was prompted by the most recent Italian guidelines on ART, issued in 2024 by the Ministry of Health in collaboration with the Italian National institute of Health (*Istituto Superiore di Sanità*) and of the National Health Council (*Consiglio Superiore di Sanità*) which, among other things, allowed posthumous reproduction through the use of cryopreserved embryos (3). Formerly characterized by restrictive reproductive policy, Italy now constitutes a significant case of how evolving medical techniques challenge established legal and ethical principles of procreation (4).

From an ethical standpoint, posthumous reproduction raises profound dilemmas. The deliberate creation of a child destined to be born without one living parent introduces tension between the adult's right to reproductive self-determination and the future child's right to relational and emotional security. While the loss of a parent may occur naturally as an accident of life, in posthumous conception it becomes the direct result of an intentional choice: the birth of a child whose father's absence is anticipated from the outset (5). This situation demands careful ethical scrutiny to ensure that the welfare of the future child is not subordinated to the psychological and existential needs of the surviving partner who wishes to pursue the pregnancy (6).

Indeed, the emotional dimension of posthumous reproduction further complicates its moral assessment. The desire to have a child with a deceased partner may stem from love, loyalty, and the wish for continuity, yet it may also intertwine with grief and difficulty in accepting the death of the loved one (5, 6). In such contexts, the new life risks being instrumentalized as a means of preserving memory or mitigating loss, contrary to the moral imperative that every person must be regarded as an end in themselves, never merely as a means to fulfill another's emotional or symbolic need. Although many such children thrive, the intentional creation of life in circumstances where parental absence is foreseen raises questions about planned vulnerability and the limits of reproductive freedom (7).

This topic requires a broad reflection that can only start by a close look at what is already happening. By comparing how different European states regulate the implantation of cryopreserved embryos in the woman's womb after the death of the male partner, this study seeks to contextualize this practice, which is not as studied and documented in academic literature as the other forms of posthumous reproduction (namely, the use of cryopreserved gametes or gametes retrieval after death).

## 2 Materials and methods

This study investigates the regulation of posthumous assisted reproduction in terms of posthumous embryo transfer, focusing specifically on: whether this practice is permitted in each jurisdiction; whether written informed consent specific to this practice is required; the temporal scope and validity of such consent; and the presence of waiting periods or other temporal limits before the procedure can be accessed.

The countries assessed were Belgium, France, Greece, Italy, Portugal, Spain, the Netherlands, and the United Kingdom.

To address these questions, the analysis draws on a range of primary and secondary sources, including national legislation, ministerial guidelines, judicial decisions, and official publications issued by national ART authorities and bioethics committees, supplemented by relevant academic literature. Only materials available in English, Italian, French, or Spanish were included to ensure accurate interpretation.

These materials were analyzed through a comparative and qualitative approach. The extracted data were synthesized, and the results are presented by country, highlighting similarities and divergences in regulatory models.

Data extraction was performed by two authors (CR, PR), and the final version of the manuscript was reviewed and approved by all contributors.

## 3 Results

Here we present a country-by-country overview of how posthumous reproduction with cryopreserved embryos is regulated across the jurisdiction included in the study. Countries are listed in alphabetical order.

### 3.1 Belgium

Belgium regulates ART through the Law of 15 March 2007 on Medically Assisted Reproduction and the Disposition of Supernumerary Embryos and Gametes (*Loi relative à la procréation médicalement assistée et à la destination des embryons surnumé-raires et des gametes*), as amended in 2023 (*Loi modifiant la loi du 6 juillet 2007 relative à la procréation médicalement assistée et à la destination des embryons surnuméraires et des gametes*) (8, 9). The statute was intended to provide legal certainty for fertility practice, given the need to take into account the variety of ethical perspectives and family structures that characterize contemporary ART practice (10, 11).

Before any treatment, each couple must sign a written agreement specifying the intended use of embryos (as well as gametes), including instructions for situations such as separation, incapacity, or the death of one partner, as well as the future of their cryopreserved material after the storage period allowed (10).

The legislation explicitly addresses post-mortem implantation: article 15 allows embryos to be used after the death of one partner if both partners had given explicit consent for such use in the original written agreement mentioned before. Indeed, Article 13 expressly requires that the consent document specifies the intended use of any cryopreserved embryos in the event of the death of one of the partners. Article 16 establishes the temporal limits for treatment: embryo transfer may take place no earlier than six months and no later than five years after the death of the male partner. The six-month period serves as a reflection interval, while the time limit—originally two years and extended to five years by the 2023 law—wishes to maintain a temporal connection with the pre-existing relationship (10).

The Belgian framework thus authorizes posthumous reproduction under conditions of documented, pre-mortem consent and defined temporal limits, and all decisions regarding embryos must be recorded in advance, with patients retaining the right to modify their directives jointly during life.

### 3.2 France

The French regulation of assisted reproduction, and particularly of posthumous conception, represents one of the most intricate and debated examples in Europe. Its legal and ethical structure is built upon a long tradition of bioethical legislation, beginning with Law n. 94-654 of July 29, 1994, on the donation and use of elements and products of the human body, medical assistance, procreation and prenatal diagnosis, and subsequently revised with the Bioethics Law in 2004, 2011, and most recently in 2021 (*Loi bioéthique*, 2004, 2011, 2021) (12–15).

Access to ART has been regulated by the Public Health Code (*Code de la santé publique*). Article L. 2141-2 of the Public Health Code as amended by the 2021 Bioethics Law, explicitly prohibits posthumous reproduction (15): it states that no medical assistance to procreation may be undertaken after the death of one of the members of the couple. This prohibition extends both to posthumous insemination and embryo transfer, and to the storage and subsequent use of gametes or embryos once one partner had died.

The 2021 Bioethics Law introduced major reforms to the French ART system, including the extension of access to single women and female couples (16). While the extension of access to ART marks a significant evolution in the social understanding of family, it did not erode the underlying conviction that reproduction after death lies beyond the ethical and legal boundaries of procreation.

Although posthumous embryo transfer remains forbidden on French territory, individual cases continue to challenge the boundaries of the law through cross-border treatment, most commonly in Spain or Belgium, where the practice is permitted with consent. However, in the recent case of Baret and Caballero v. France, the European Court of Human Rights (ECHR) upheld France's ban on posthumous reproduction, ruling that the prohibition did not violate the right to private and family life under Article 8 of the European Convention on Human Rights (17). The ECHR held that the ban imposed by French law, which prevents the export of gametes and embryos for posthumous reproduction, was legitimate because it pursued legitimate aims, namely the “protection of morals” and “the protection of the rights and freedom of others”: these are concepts that in ECHR case law allow States to regulate sensitive ethical issues and to safeguard the interests of third parties potentially affected by decisions. The ECHR concluded that France enjoys a wide margin of appreciation in making such ethical and social policy choices (18).

### 3.3 Greece

In the Greek legal framework, the principal sources are Law 3089/2002 on Medical Assistance in Human Reproduction (*Ιατρική υποβοήθηση στην ανθρώπινη αναπαραγωγή*) and Law 3305/2005 on the Application of Medically Assisted Repro-duction Methods (*Εφαρμογή της Ιατρικώς Υποβοηθούμενης Αναπαραγωγής*) (19, 20): the first law mainly replaces the relevant articles of the Greek Civil Law Code, and the second law is complementary to the first one, clarifying some issues and established the National Authority of Medically Assisted Reproduction (21). These instruments define the conditions of access, the forms of consent required, and the ethical boundaries of reproductive practice, and address sensitive issues such as donor anonymity, surrogacy, embryo research, and post-mortem reproduction (21).

According to the law of 2005, when natural procreation cannot occur due to infertility or the risk of transmitting a disease to the offspring, ART is deemed as a medical necessity. Access to assisted reproduction is granted not only to married couples but also to unmarried or cohabiting partners and to single women up to the age of fifty-two. Each procedure must be preceded by explicit written and informed consent, and the parties must determine in advance the fate of any embryos created, particularly in circumstances such as divorce, separation, or death (21).

Article 1,457 of the Civil Code, introduced by Law 3089/2002, allows a woman to proceed with fertilization using the gametes of her deceased husband or partner, as well as the embryo created with him, provided that two cumulative conditions are met. First, there must be prior written consent, expressed in a notarial act, in which the deceased explicitly authorizes the posthumous use of his sperm or embryos created during life. Second, the law imposes a temporal limitation: the procedure may take place no earlier than six months and no later than two years after the man's death (21). The waiting period is intended to ensure reflection, while the time limit reflects an intention to preserve the connection between conception and the existence of a recent, living relationship. Greece allows posthumous reproduction only when clearly premeditated, temporally constrained, and legally valid.

### 3.4 Italy

The country's ART framework is governed by Law n. 40 of 19 February 2004 on Medically Assisted Procreation (*Norme in materia di procreazione medicalmente assistita*), a law that sought to protect “all subjects involved, including the conceived,” while setting clear limits to the use of reproductive technologies (22). Initially, the law was among the most restrictive in Europe, but over the following two decades, constitutional jurisprudence and ministerial revisions progressively reshaped the legal landscape (Constitutional Court rulings 2009, 2014, 2015) allowing more and more applicability (23–26).

A recent development of the regulation was shaped by the 2024 Guidelines for ART, issued by the Ministry of Health in collaboration with the Italina National institute of Health (*Istituto Superiore di Sanità*) and of the National Health Council (*Consiglio Superiore di Sanità*), which introduced substantial updates to the interpretation and ap-plication of Law 40/2004 (3). Among the most significant provisions is the possibility of post-mortem embryo transfer (5.4, Guidelines 2024) (3).

These provisions aligned national practice with recent constitutional and judicial reasoning, which emphasized the enduring significance of informed consent as the determining factor in the creation of parental bonds, as expressed already in Article 8 of Law 40/2004 (Cassation Court ruling 13,000/2019, point 7.8.5.1) (27). The guidelines reaffirmed that once consent has been given and fertilization has occurred, it becomes irrevocable (Art. 6, Law 40/2004)—reflecting the Italian legal understanding of ART as a process initiated by a shared and deliberate reproductive project (4, 6).

A seven-day waiting period must elapse between the signing of consent and the fertilization of the oocyte, allowing time for reflection and ensuring that the decision is informed and deliberate; this waiting time between consent and fertilization has been in place since the law's enactment. Physicians and healthcare providers are required to provide comprehensive information to the couple, including the relevant bioethical implications. The 2024 Guidelines do not impose any temporal limitation on the post-humous use of embryos.

### 3.5 Portugal

The Portuguese framework for medically assisted reproduction is established by Law 32/2006 on medically assisted reproduction (*Procriação medicamente assistida*), as amended (28). It regulates access to treatment, donation, cryopreservation, embryo re-search, and other aspects of assisted reproduction.

Under Article 22, posthumous embryo transfer is permitted in order to realize a clearly defined and previously consented “parental project”, with no specific mention to a dedicated consent to posthumous use of embryos (29). At the same time, the law specifies that when consent is given by the couple, they should be informed of all benefits and risks embedded in the procedure, as well as their ethical, social and legal implications (Art 14.2). The law provides that the woman can proceed with the embryo transfer after a period considered appropriate for proper reflection and consideration (Art 22.1), a period deemed to be not less than six months, except for critical clinical reasons that will be certified by the physicians overseeing the whole procedure (Art 22.4). The embryo transfer must be done within three years of the partner's death. The article also specifies that psychological support must be made available before, during, and after the process.

### 3.6 Spain

In Spain, the legal framework is found primarily in Law 14/2006, on Human Assisted Reproduction Techniques (*Sobre técnicas de reproducción humana asistida*) and Law 14/2007 on Biomedicine (*de Investigación biomédica*) (30, 31).

Article 9 of the 2006 law first states that no legal parentage or relationship may be recognized for those children of a man who has died before “his reproductive material” was already in the woman's uterus (32). However, in the following paragraph, the law allows for posthumous embryo transfer when the man has given specific consent for his reproductive material—a thus for their existing embryos—to be used by his wife. This consent must be documented in one of the legally recognized forms: a public deed, a will, or a document of anticipated directives, or the clinical consent document referred to in the same law. The law also sets a temporal limit of twelve months from the date of death within which embryo transfer must take place (Article 9.2) (30).

In the case of unmarried partners, Article 9.3 extends the same possibility: a man who is not married to the woman undergoing treatment may authorize, under the same formal requirements, the posthumous use of his reproductive material (30).

### 3.7 The Netherlands

The Dutch approach to posthumous reproduction is governed by a combination of statutory provisions under the Embryo Act (*Embryowet*) issued in 2002 and detailed professional guidelines issued by medical associations (33). While the Embryo Act itself does not explicitly regulate posthumous reproduction, it establishes general requirements for consent and the lawful use and storage of embryos. Specifically, Article 7 of such Act stipulates that reproductive material must be destroyed upon the individual's death, unless written consent for posthumous storage and use was given during their lifetime.

The legal framework is complemented by professional standards contained in the Guidelines of the Embryo Act (*Modelreglement Embryowet*), jointly issued by the Dutch Society of Obstetrics and Gynecology (*NVOG*) and the Association for Clinical Embryology (*KLEM*)—first in 2004 and then updated in 2018 (34). The 2018 Guidelines provides a structured, practice-oriented interpretation of the Embryo Act and is considered binding on fertility clinics as a professional norm. Chapter 5 addresses post-humous reproduction, defined as any conception occurring after the death of one or both genetic contributors, whether through stored gametes or embryos (34).

Under these guidelines, posthumous use of reproductive material is only permissible where the explicit, prior, and written consent by the deceased exists. In the absence of such consent, retrieval and use are not authorized, and clinics are instructed to destroy the material once the provider's death is confirmed.

The Guidelines also outlines procedural and ethical recommendations: first of all, a twelve-month reflection period is advised between the death of the gamete provider and the initiation of fertility treatment using the material. This is because there is a psychological instability that follows a partner's death, and although it is very personal, the regulation claims that after twelve months it is clear whether the grieving process has come to a resolution (*Embryowet*, 5.5) (34). During this time, psychological counselling should be offered. The time limit for the procedure is the time limit in terms of age for all women to access the procedure, which is 49 years old.

### 3.8 The United Kingdom

The United Kingdom represents one of the most comprehensive and precisely regulated systems of assisted reproduction in Europe. Its framework is defined by the Human Fertilisation and Embryology Act 1990 (Human Fertilisation and Embriology act 1990), as amended by the Human Fertilisation and Embryology Act 2008 (Human Fertilisation and Embriology act 2008) and implemented through the work of the Human Fertilisation and Embryology Authority (HFEA) (35, 36). The HFEA, established in 1991, acts as the national independent regulator for fertility clinics and embryo research. Its authority extends to licensing, monitoring compliance, and ensuring that the use and storage of gametes and embryos occur only with proper consent and within clear temporal and ethical boundaries.

At the core of the UK system is the principle of informed, written, and specific consent. Before any treatment or storage can occur, couples must complete legally prescribed HFEA forms specifying how their embryos may be used, for how long, and under what circumstances. In the UK, consent can be withdrawn at any time up until an embryo is transferred into the womb; if an individual wishes for his partner to use their embryos after his death, he should name his partner on the specific consent form. The HFEA requires physicians to ensure that individuals fully understand the implications of their decisions and to offer counselling prior to consent.

The regulatory framework explicitly addresses posthumous use of gametes and embryos. The law allows for the use and storage of reproductive material after death, provided that the deceased has given explicit written consent prior to their death. This consent must specify both the use and continued storage of embryos after death and name the partner who may use them. Without this explicit authorization, posthumous use is prohibited, and any embryos in storage must be destroyed once they can no longer be lawfully held (37).

According to the HFEA guidance (Consent to Treatment and Storage, updated 2022), where valid consent has been given, embryos may remain stored for up to 10 years following the individual's death, exclusively for use by the partner named on the specific consent form. After this period, they must be removed from storage and disposed of. The deceased may also consent to shorter storage periods, or to the use of their embryos for training or research purposes after death. The system relies on the clarity and validity of consent forms, which function as legal instruments of posthumous intention.

## 4 Discussion

The comparative overview reveals a heterogeneous European landscape regarding posthumous embryo transfer. Although the practice is allowed in all countries considered except France, their regulatory approaches vary. France, indeed, maintains a strict prohibition, a position that has been repeatedly upheld both at the domestic level and by the European Court of Human Rights in Baret and Caballero v. France.

Among the countries that allow posthumous embryo transfer, Italy and Portugal do not require a specific form of consent for post-mortem use of embryos; instead, they consider the couples’ prior consent to the shared parental project to suffice in this scenario as well. In contrast, Belgium Greece, Portugal, the Netherlands, and the United Kingdom allow the practice when the deceased partner has provided explicit, written, and pre-mortem authorization.

Jurisdictions that require explicit, written, and pre-mortem consent from the deceased partner (Belgium, Greece, Spain, the Netherlands, and the United Kingdom) place greater emphasis on individual self-determination. By contrast, systems that consider the couple's prior consent to embryo creation within a joint reproductive plan as sufficient give priority to the shared nature of the parental intention and to the continuity of the couple's reproductive project.

A further point of divergence concerns to temporal limitations: Belgium, Greece and Portugal require a minimum waiting period of six months, although Portugal frames it as a recommendation that may be waived for clinically justified reasons. The Netherlands, instead, does not impose a legal limit but recommends, through professional guidelines, a twelve-month reflection period. Italy, Spain and the United Kingdom do not require—nor do they recommend—any waiting time before the surviving partner proceeds with embryo transfer.

As for the maximum time within which the procedure may be performed, Spain—despite not mandating a reflection time—grants a twelve-month window for posthumous embryo transfer. Greece allows the practice up to two years after the partner's death, Portugal up to three years, and Belgium up to five years. The United Kingdom permits posthumous embryo transfer up to ten years after death unless the deceased partner's written consent specifies a shorter period. The Netherlands sets the woman's age limit of 49 years as the operative boundary (corresponding to the general ART eligibility threshold), while Italy imposes no temporal restriction, provided that consent was given before death and fertilization occurred during the partner's lifetime.

These temporal discrepancies reveal that European systems do not share a common understanding of whether and how time should operate as a safeguard in posthumous reproduction. Some jurisdictions insist on waiting periods or maximum time limits, while others deliberately refrain from introducing them. This divergence reflects different ways of conceiving the nature of the reproductive project and the extent to which its continuation after death should remain anchored to the period in which both partners were alive.

While regulatory frameworks focus primarily on consent, temporal limits can be understood as an indirect way of addressing the best interests of the future child, as well as the stability of the mother. As highlighted by various legal frameworks and professional guidelines in those countries that regulate a waiting or reflection period, this interval—ranging from six to twelve months—serves to promote emotional stability and to ensure that the decision does not stem from an immediate or grief-driven impulse (6, 38, 39). In this sense, waiting periods can be understood as a safeguard to reduce the risk that their birth functions as a compensatory response to a loss rather than as a considered continuation of a reproductive project.

At the same time, by avoiding excessively delayed conceptions, several jurisdictions aim to ensure that the child is not born into circumstances too far removed from the lived relational context of the parental project, and from the lived experience of their already deceased father. The underlying rationale is that prolonged temporal distance may weaken the symbolic and social connection between the child and the deceased father, potentially complicating identity formation and familial integration (6, 38, 39). In this sense, maximum temporal limits can be also be understood as a safeguard for the prospective well-being of the child.

The presence or absence of temporal safeguards is normatively significant: systems that combine consent requirements with some temporal constraints appear better equipped to balance reproductive autonomy with ethical and social considerations, particularly regarding the prospective welfare of the child. By contrast, frameworks that refrain entirely from temporal regulation risk marginalizing child-centered considerations.

This tension lies at the core of the complexity generated by ART. Lawmakers and society are increasingly confronted with scenarios that challenge traditional categories and assumptions, and that resist straightforward normative solutions, at times giving rise to regulatory frameworks that are difficult to reconcile even internally. This emerges with clarity in the Italian case, where a legal framework strongly oriented towards embryo protection does not address the temporal and relational implications of posthumous reproduction.

## 5 Conclusions

This work set out to explore the ways in which different European jurisdictions regulate posthumous embryo transfer, especially regarding consent and the temporal conditions attached to its use.

The comparative overview shows that, while the practice is permitted in most of the countries examined, the underlying regulatory frameworks differ significantly. The main divergences concern the form and scope of consent—ranging from reliance on general ART consent to explicit, written, pre-mortem authorization—and the temporal conditions under which posthumous embryo transfer may take place. Some jurisdictions impose waiting periods or maximum timeframes to preserve a symbolic and relational continuity with the original parental project, while others do not introduce any temporal restriction at all.

These findings highlight that European countries do not share a uniform understanding of how posthumous reproduction should be regulated, and that each legal system interprets the balance between reproductive autonomy, procedural safeguards, and the prospective welfare of the child in distinct ways.

The present study has some limitations: first, not all European jurisdictions were included, and the analysis focused only on those countries for which relevant legal sources and academic commentary were accessible; some documents may not have been retrieved or may not have been publicly available at the time of writing. Moreover, the study relied on materials available in English, Italian, French and Spanish: the absence of sources in other languages may have led to an incomplete understanding of some national frameworks.

Future research would benefit from extending this analysis to extra-European legal systems, in order to explore whether similar patterns emerge beyond Europe and to provide a broader understanding of the regulatory and ethical challenges posed by posthumous reproduction. It is our intention to pursue this line of inquiry in subsequent work.
